# Book Review: Computational Toxicology. Risk Assessment for Pharmaceutical and Environmental Chemicals, 2nd Edition

**DOI:** 10.3389/fphar.2019.00274

**Published:** 2019-03-26

**Authors:** Marcelo T. de Oliveira

**Affiliations:** São Carlos Institute of Chemistry, São Paulo University, São Carlos, Brazil

**Keywords:** prediction, toxicology, QSAR, big data, artificial intelligence

After a long day, I was browsing on my Instagram account and there it was among stories, the cover of *Computational Toxicology* new edition, posted by a colleague and co-author to one of the chapters. Back in 2007, when the original volume was launched, this social media platform didn't even exist. Technology is impacting our lives in so many ways, and unarguably also rapidly transforming the way we do science. A decade on, the editor has produced a major makeover to the title to reflect the tech advances on the topic marked by collaborative initiatives such as e-Tox and OpenTox as well as model sharing and various new computational tools, which are now often open source. As in so many areas, the explosion of data been made available publicly in recent years represents the game changer. The new edition is cut for the next generation of computational toxicologists starting to deal with the challenges in the era of “big data.”

If the first edition could be considered lengthy stretching over 800 pages. It has got considerably leaner to about half of the original size. The book is now divided into 4 parts instead of 5 as a dedicated introductory part to general toxicology methods has been removed. The reader now opens up the book straight into computational methods including dedicated sessions to methods like deep neural networks, random forest, decision tree, and so forth, which were not to be found previously or only quickly mentioned producing an updated, to-the-point text. For all these methods and throughout the book embedded in the text, the central issue of method validation is presented and discussed in various contexts. Still in part I (Computational Methods), a chapter on quantum mechanical approaches in computational toxicology has been introduced.

In Part II—Applying Computers to Toxicology Assessment: Pharmaceutical, Industrial and Clinical, various chapters centered around methods such as QSAR, homology modeling, or crystal structures in the previous edition, now give way to a problem-based organization over a wide range of topics on computational applications from *Predicting hERG Activity, Traditional Chinese Medicine* to the *Clinical Setting*. Part III, which also deals directly with applications in the Environmental arena, now also includes Regulatory. In these two parts lies the cream of the book offering the reader a better appreciation of the state-of-the-art of applications in the field while also faced with new concepts such as *toxicity cliff*, *public chemotype approach* or the use of toxicology apps in mobile devices following many of the trends in other areas particularly medicinal chemistry. Certainly, inspiration can be drawn from the reading of chapters in both parts.

The book comes to a closure as in the previous edition with a few chapters on future perspectives for computational toxicology. From the first edition, very few if anyone at all could have anticipated how fast the field would have evolved to what it is turning into. Computational toxicology is unarguably maturing to an increasingly more relevant field on its own merits responding boldly to problems in multiple areas—that is, delivering computer-based predictions. Furthermore, by being more integrated to medicinal chemistry, biochemistry, pharmacology, and organic chemistry among other subjects attracts the right attention contributing to its visibility. What lies ahead for the next decade can perhaps be better forecasted this time. The final chapters focus on the continuing challenges of “big data” in the field, the prospects on new computational methods, open-data repositories for toxicology and opportunities in predicting adverse drug adverse effects.

Once again, the title represents a major contribution to the field from a wide international group of authors, and this new edition surely lives up to the expectation. A must-have reference for experts and non-experts alike.

## Author Contributions

The author confirms being the sole contributor of this work and has approved it for publication.

### Conflict of Interest Statement

The author declares that the research was conducted in the absence of any commercial or financial relationships that could be construed as a potential conflict of interest.

